# The immediate effect of a soft knee brace on pain, activity limitations, self-reported knee instability, and self-reported knee confidence in patients with knee osteoarthritis

**DOI:** 10.1186/s13075-017-1456-0

**Published:** 2017-12-01

**Authors:** Tomasz Cudejko, Martin van der Esch, Marike van der Leeden, Josien C. van den Noort, Leo D. Roorda, Willem Lems, Jos Twisk, Martijn Steultjens, James Woodburn, Jaap Harlaar, Joost Dekker

**Affiliations:** 1 Department of Rehabilitation Medicine, VU University Medical Center, Amsterdam, The Netherlands; 2 Amsterdam Rehabilitation Research Centre Reade, Dr. J. van Breemenstraat 2, 1056 AB Amsterdam, P.O. Box 58271, 1040 HG Amsterdam, The Netherlands; 3 Department of Radiology and Nuclear Medicine, Musculoskeletal Imaging Quantification Center (MIQC), Academic Medical Center, Amsterdam, The Netherlands; 4 Amsterdam Rheumatology & Immunology Center, Amsterdam Medical Center, Reade, VU University Medical Center, Amsterdam, The Netherlands; 5 Jan van Breemen Research Institute, Reade, Amsterdam, The Netherlands; 6 Department of Health Sciences, VU University Amsterdam, Amsterdam, The Netherlands; 7 Department of Epidemiology and Biostatistics, VU University Medical Center, Amsterdam, The Netherlands; 8 School of Health and Life Sciences, Glasgow Caledonian University, Glasgow, United Kingdom; 9 Delft University of Technology, Delft, The Netherlands; 10 Department of Psychiatry, VU University Medical Center, Amsterdam, The Netherlands

**Keywords:** Knee, Osteoarthritis, Brace, Orthotics

## Abstract

**Background:**

We aimed to (i) evaluate the immediate effect of a soft knee brace on pain, activity limitations, self-reported knee instability, and self-reported knee confidence, and (ii) to assess the difference in effect between a non-tight and a tight soft brace in patients with knee osteoarthritis (OA).

**Methods:**

Forty-four patients with knee OA and self-reported knee instability participated in the single-session, laboratory, experimental study. A within-subject design was used, comparing a soft brace with no brace, and comparing a non-tight with a tight soft brace. The outcome measures were pain, self-reported knee instability and knee confidence during level and perturbed walking on the treadmill and activity limitations (10-m walk test and the get up and go (GUG) test). Linear mixed-effect model analysis for continuous outcomes and logistic generalized estimating equations for categorical outcomes were used to evaluate the effect of wearing a soft brace.

**Results:**

Wearing a soft brace significantly reduced pain during level walking (*B* − 0.60, *P* = 0.001) and perturbed walking (*B* − 0.80, *P* < 0.001), reduced the time to complete the 10-m walk (*B* − 0.23, *P* < 0.001) and the GUG tests (*B* − 0.23, *P* = 0.004), reduced self-reported knee instability during level walking (OR 0.41, *P* = 0.002) and perturbed walking (OR 0.36, *P* < 0.001), and reduced lack of confidence in the knees during level walking (OR 0.45, *P* < 0.001) and perturbed walking (OR 0.56, *P* < 0.001), compared with not wearing a soft brace. There was no difference in effects between a non-tight and tight brace, except for the 10-m walk test. Wearing a tight brace significantly reduced the time to complete the 10-m walk test in comparison with wearing a non-tight brace (*B* − 0.11, *P* = 0.03).

**Conclusion:**

The results of this study indicate that a soft brace is an efficacious intervention targeting pain, activity limitations, self-reported knee instability, and knee confidence in the immediate term in patients with knee OA. Further studies are needed evaluating the mode of action based on exerted pressure, and on the generalization to functioning in daily life.

**Trial registration:**

trialregister.nl, NTR6363. Retrospectively registered on 15 May 2017.

## Background

Soft braces are elastic, non-adhesive orthoses recommended in the non-surgical management of patients with knee osteoarthritis (OA) [1]. Because of ease of use, lack of complications, and low cost, soft braces are commonly used with the aim of reducing pain and activity limitations [1]. Soft brace efficacy and effectiveness has been assessed in laboratory [2, 3] and ambulatory settings [4, 5]. A laboratory setting allows the assessment of the immediate efficacy of an intervention under controlled conditions, while an ambulatory setting allows the assessment of the effectiveness in real life, where uncontrollable factors may be present. The evidence for immediate efficacy of a soft brace in patients with knee OA is limited. A recent systematic review with meta-analyses on soft bracing in knee OA showed an immediate, moderate improvement in pain when wearing a soft brace [6]. As the results were based on only two studies of low to moderate quality, however, further studies are needed.

It is not well-understood how a soft brace might reduce activity limitations and pain in patients with knee OA. A decrease in pain might be due to sensory stimulation of the skin, leading to a reduction in transmission of pain signals [7]. It is also assumed that a soft brace stimulates cutaneous mechanoreceptors, resulting in improved joint proprioception accuracy, which may have an impact on knee joint stability [2]. Improved knee joint stability might reduce activity limitations [8, 9]. To our knowledge, there is only one study that showed that a soft brace improves self-reported knee instability [3], while the effect of a soft brace on instability-related lack of confidence in the knee [10] is unknown. Externally applied perturbations challenge knee stabilization strategies in patients with knee OA [11, 13]. Perturbed conditions therefore provide a powerful test of soft braces. However, whether a soft brace has an effect on knee stability and knee confidence in perturbed conditions has not been assessed yet. Given the high prevalence of self-reported knee instability and lack of confidence in the knee in patients with knee OA [9, 14], there is a need to study the effect of a soft brace on knee stability and knee confidence during both level and perturbed walk.

Hassan et al. [15] reported that a non-tight soft brace was more effective than a tight brace in reducing pain and improving postural sway in patients with knee OA. The authors suggested that a non-tight brace provides more recurrent stimuli to cutaneous mechanoreceptors, whereas a tight brace provides constant pressure, to which skin mechanoreceptors adapt [15]. There is a clear need to replicate this study in order to determine whether the level of tightness has an effect on activity limitations, knee stability and knee confidence.

The currently available evidence for the use of soft braces in patients with knee OA is limited and of low quality. Thus, the aims of the study were to (i) evaluate the immediate effect of a soft brace on pain, activity limitations, and self-reported knee instability and knee confidence, and to (ii) assess the difference in effect between a non-tight and a tight soft brace in patients with knee OA. We hypothesized that (i) a soft brace reduces pain, activity limitations, and self-reported knee instability, and improves knee confidence, and that (ii) a non-tight soft brace has a greater effect than a tight soft brace.

## Methods

### Trial design

In this single-session, laboratory, experimental study, a within-subject design was used, comparing a soft brace with no soft brace, and comparing a non-tight with a tight soft brace. The order of the non-tight and tight brace was randomized, using a computer generated random sequence made prior to the study. All participants were blinded to the type of brace (non-tight or tight).

### Participants

Participants were recruited from the Amsterdam Osteoarthritis (AMS-OA) cohort through telephone-based screening between August 2015 and April 2016. The AMS-OA cohort consists of participants with established knee and/or hip OA according to the American College of Rheumatology (ACR) criteria [16], who had been referred to an outpatient rehabilitation center (Reade, Center for Rehabilitation and Rheumatology, Amsterdam, the Netherlands). Participants were assessed by rheumatologists, radiologists, and rehabilitation physicians. According to the ACR criteria, knee OA is diagnosed if knee pain is present and three of the following six parameters are present: age > 50 years, morning stiffness < 30 minutes, crepitus, bony tenderness, bony enlargement and no palpable warmth. The inclusion criteria for the present study were (i) diagnosis of bilateral or unilateral knee OA according to the ACR criteria [16], (ii) age between 50 and 80 years, and (iii) presence of self-reported knee instability in the past 3 months. Self-reported knee instability was defined as at least one episode of buckling, shifting, or giving way of the knee in the last three months, reported by participants [17].

The exclusion criteria for the AMS-OA cohort were total knee replacement and/or inflammatory arthritis (including rheumatoid arthritis, crystal arthropathy, septic arthritis, and spondylarthropathy). Additional exclusion criteria for the present study were (i) radiographic patello-femoral (PF) joint OA (PFOA), and (ii) presence of comorbidity resulting in severe activity limitations. The soft brace in the current study did not involve patellar opening, thus, to avoid applying pressure on the PF compartment, we excluded patients with PFOA.

### Interventions

A commercially available soft brace (GENUTEX A2, Human I; Bleiswijk, The Netherlands) was used. The following sizes of the soft brace were available: S: 30–35 cm, M: 35–40 cm, L: 40–45 cm, XL: 45–50 cm, and XXL: 50–55 cm. These sizes were used to differentiate between a non-tight and tight soft brace. A tight brace was defined as one fitted based on shank and thigh circumferences measured according to instructions provided by the distributor. A non-tight brace was defined as one a size larger than a tight brace. Fitting and positioning of the brace were performed by a trained investigator (TC) according to instructions provided by the distributor. Both knees were braced regardless of the knee OA status. Participants were allowed to become accustomed to the brace before treadmill walking. Participants were blinded to the type of brace by informing them that the study was a comparison of two different braces, without mentioning differences in tightness. Braces were identical in appearance.

### Primary outcome measure

#### Pain

Knee pain during walking on the treadmill was assessed using the Dutch-translated 11-point numerical rating scale (NRS) (0–10), with higher scores representing more pain [18]. The following question was asked during both level and perturbed walking: “On a scale from 0 to 10, how would you score the level of your left/right knee pain while walking on the treadmill?” The Dutch translated NRS has been frequently used in the Netherlands [17, 19, 20].

### Secondary outcome measures

#### Activity limitations

Activity limitations were assessed with two standardized physical performance tests: the 10-m walk test [21] and the get up and go (GUG) test [22]. The 10-m walk test assesses the time to walk a distance of 10 m along a level and unobstructed corridor [21]. Participants were instructed to walk as fast as possible and timed with a stopwatch. The GUG test measures the time a person takes to get up from a chair and walk 15 m as fast as possible along a level and unobstructed corridor [23]. The intraclass correlation coefficient (ICC) for the GUG test is 0.98 for intra-tester reliability and 0.98 for inter-tester reliability [22].

#### Self-reported knee instability

Self-reported knee instability was assessed with the Dutch version of the questionnaire developed by Irrgang et al. [24]. Self-reported knee instability was assessed with a question on the number of episodes during walking on the treadmill of perceived buckling, shifting, or giving way, separately for the right and left knee. The following question was asked during both level and perturbed walking: “How many times have you had a sensation of left/right knee buckling, shifting, or giving way while walking on the treadmill?” Self-reported knee instability was dichotomized into no episode of knee instability versus one or more episodes of knee instability. The translated Dutch version of the questionnaire has been previously used by our research group [20, 25].

#### Self-reported knee confidence

Knee confidence was assessed using a 5-point Likert scale (not at all, mildly, moderately, severely, and extremely) in response to the question asked during both level and perturbed walking: “How much were you troubled with lack of confidence in your left/right knee while walking on the treadmill?” This is a single item from the Dutch-validated Knee injury and Osteoarthritis Outcome Score (KOOS) [26–28]. Self-reported knee confidence was dichotomized into lack (mildly, moderately, severely, or extremely troubled) versus full (not troubled at all) knee confidence.

### Other measures

Participants’ demographic and clinical characteristics were recorded prior to testing and included: age, gender, duration of symptoms, body-mass index (BMI), knee OA radiographic severity, and muscle strength. BMI was calculated as body mass in kilograms divided by height in square meters. The Kellgren and Lawrence score (KL score) from the more severely affected knee was used to assess the radiographic severity of the disease [29]. Conventional radiographs of tibiofemoral joints were made by a weight-bearing posterior-anterior view according to Buckland-Wright et al. [30]. Two independent observers graded radiographs: a bone and joint radiologist and an epidemiologist trained by two musculoskeletal radiologists. Muscle strength (in Newton meters per kilogram of body weight (Nm/kg)) was assessed for flexion and extension of the knee using an isokinetic dynamometer (Humac Norm, CSMi, Boston, USA) according to a previously described protocol [11]. The mean strength of the quadriceps and hamstring muscles of the left and right knee was reported. The Dutch translation of the Western Ontario and McMaster Universities Osteoarthritis Index (WOMAC) was used to assess self-reported knee pain, stiffness, and activity limitations of the most affected knee [12, 13].

### Sample size

The study was powered on self-reported pain. An expected effect size of 0.5 was calculated from the change in pain and its confidence interval reported in the study of Hassan et al. [15]. With effect size = 0.5, alpha = 0.05, power (1-β) = 0.80, and a two-sided test, the minimum number of subjects required was n = 33. Allowing for an attrition rate of 10% during the course of the study [31], this study needed to include at least 36 patients.

### Procedure

The study procedure is presented in Table 1. Participants of the study attended a single testing session. Outcome measures, with the exception of the 10-m walk and GUG tests, were assessed on the treadmill, which is integrated into the GRAIL system. The GRAIL system is made up of a treadmill with a dual belt, placed in a virtual reality environment (GRAIL system, MOTEKForce Link, The Netherlands). Walking on the treadmill was initially performed with no brace. Patients had a familiarization session on the treadmill lasting at least one minute, during which walking speed was determined. They were instructed to walk at a comfortable, self-selected pace while shod and wearing a safety harness. Following the familiarization session, participants were subjected to two tasks (without wearing a brace): (i) level walking for 2 minutes and (ii) walking with mechanical perturbations on the treadmill. Mechanical perturbations on the treadmill comprised five lateral-medial translations (2 cm displacements) of the treadmill belts during the stance phase of a gait cycle. For safety reasons, participants were informed about mechanical perturbations in advance. The mechanical perturbations of the treadmill occurred at random moments. Immediately following each of the two tasks, participants reported their average levels of knee pain, number of knee instability episodes, and perceived knee confidence. Subsequently, participants were randomized to receive a non-tight or tight brace and the two tasks were repeated while wearing a brace. Prior to testing on the treadmill, the 10-m walk and GUG tests were performed without the brace. Following testing on the treadmill, the 10-m walk and GUG tests were repeated with the brace (non-tight or tight).

**Table 1:** Study procedure

Baseline assessment without an SB	Intervention assessment with an SB^a^	Rest^b^	Baseline assessment without an SB	Intervention assessment with an SB^a^
1. 10-m walk test 2. GUG test 3. Level walk A. Pain B. SKI and SCI 4. Perturbed walk A. Pain B. SKI and SCI	1. Level walk A. Pain B. SKI and SCI 2. Perturbed walk A. Pain B. SKI and SCI 3. 10-m walk test 4. GUG test		1. 10-m walk test 2. GUG test 3. Level walk A. Pain B. SKI and SCI 4. Perturbed walk A. Pain B. SKI and SCI	1. Level walk A. Pain B. SKI and SCI 2. Perturbed walk A. Pain B. SKI and SCI 3. 10-m walk test 4. GUG test

*SB* soft brace, *SKI* self-reported knee instability, *GUG* get up and go test, *SCI* self-reported knee confidence

^a^Tight or non-tight soft brace

^b^Rest of 30 minutes

After a 30-minute rest, the procedure crossed over to the second part of the assessments but with another type of soft brace (tight or non-tight). By counterbalancing the order of the type of brace we controlled the potential confounding effect of fatigue and learning effects on the comparison between a non-tight versus tight brace.

### Statistical analysis

Descriptive statistics were used to characterize the study population. Numbers (percentages) were used for categorical variables, and means (SD) for continuous variables. Prior to the statistical analysis, outcome measures were checked for normality using the Shapiro–Wilk and Kolmogorov–Smirnov tests. Pain and self-reported knee instability and knee confidence were obtained for each knee separately (knee-level variable), and data from both knees were used in the statistical analysis. Data on the 10-m walk and GUG tests were analyzed as person-level variables. Four comparisons were analyzed: (i) brace versus no brace, (ii) tight brace versus no brace (i.e., baseline before tight), (iii) non-tight brace versus no brace (i.e., baseline before non-tight), and (iv) non-tight brace versus tight brace. Linear mixed-effect model analysis for repeated measurements within participants was used for continuous outcome measures (pain, 10-m walk test, GUG test). Logistic generalized estimating equations (GEE) analysis with an exchangeable working correlation matrix was used for dichotomous outcome measures (self-reported knee instability and knee confidence). No covariates were included in the models, because addition of a covariate to a repeated measures analysis does not alter the main effects of a within-subject factor (e.g., age, gender, etc. are the same at each data point) [32]. Statistical significance was accepted at *P* < 0.05. All analyses were performed using SPSS software, version 22.0 (SPSS, Chicago, IL, USA).

## Results

There were 214 persons from the AMS-OA cohort initially contacted for eligibility; 144 persons declined to participate and 26 did not meet the inclusion criteria for the following reasons: absence of self-reported knee instability in the last 3 months (n = 13), comorbidity severely affecting daily functioning (n = 8), or age not between 50 and 80 years (n = 5). In total, 44 patients with knee OA and self-reported knee instability participated in the study. Demographics and clinical characteristics of the participants are shown in Table 2. The participants had a mean ± SD age of 65.7 ± 9.3 years, a mean ± SD BMI of 29.8 ± 5.5 kg/m^2^, and 29 (65.9%) were women.

**Table 2:** Descriptive statistics of the study participants (n = 44)

Variable	Value
Age (years)	65.7 (9.3)
Female, number (%)	29 (65.9)
Body mass index (kg/m^2^)	29.8 (5.5)
Duration of symptoms (years)	13.0 (10.3)
Pain last week (NRS, range 0–10)	4.7 (1.9)
WOMAC, pain (range 0–20)	8.8 (4.0)
WOMAC, stiffness (range 0–8)	4.6 (1.7)
WOMAC, physical function (range 0–68)	32.5 (12.7)
WOMAC, total score (range 0–96)	45.9 (17.5)
Muscle strength, (Nm/kg)	0.88 (0.33)
Walking speed on the treadmill, (m/s)	0.74 (0.24)
KL grade, number (%)	
0 (none)	3 (7.0)
1 (doubtful)	16 (37.2)
2 (mild)	9 (20.9)
3 (moderate)	10 (23.3)
4 (severe)	5 (11.6)
Self-reported knee instability < 3 months, number (%)	
Rarely (1–2 times)	13 (29.5)
Regularly (3–4 times)	18 (40.9)
Very often (>4 times)	13 (29.5)

Categorical variables are presented as numbers (percentage); continuous variables are presented as means ± SD

*NRS* numeric rating scale, *WOMAC* Western Ontario and McMaster Universities Osteoarthritis Index *KL* Kellgren–Lawrence score

Descriptive data on pain and activity limitations by the conditions are presented in Table 3. The results of the statistical evaluation of the outcomes are presented in Table 4. Wearing a soft brace significantly reduced pain during level walking (*B* − 0.60, *P* = 0.001) and perturbed walking (*B* − 0.80, *P* < 0.001), reduced the time to complete the 10-m walk test (B − 0.23, *P* < 0.001) and GUG test (*B* − 0.23, *P* = 0.004), reduced self-reported knee instability during level walking (OR 0.41, *P* = 0.002) and perturbed walking (OR 0.36, *P* < 0.001), and reduced lack of confidence in the knees during level walking (OR 0.45, *P* < 0.001) and perturbed walking (OR 0.56, *P* < 0.001), compared with not wearing a soft brace (Table 4, column 2).

**Table 3:** Pain and activity limitations by the conditions, mean (SD)

Outcomes	No brace	Brace	No brace	Tight brace	No brace	Non-tight brace	Tight brace	Non-tight brace
Pain, level walk^a^	3.28 (2.8)	2.67 (2.7)	3.44 (3.0)	2.83 (2.7)	3.14 (2.7)	2.53 (2.7)	2.79 (2.7)	2.55 (2.7)
Pain, perturbed walk^a^	3.41 (2.9)	2.60 (2.8)	3.51 (2.9)	2.76 (2.9)	3.33 (2.9)	2.47 (2.6)	2.72 (2.9)	2.50 (2.6)
10-m walk test^b^	7.04 (1.8)	6.81 (2.0)	6.89 (1.7)	6.67 (1.9)	7.11 (2.0)	6.89 (2.1)	6.75 (1.9)	6.86 (2.1)
GUG test^b^	10.89 (3.2)	10.66 (3.6)	10.65 (2.8)	10.57 (3.5)	11.13 (3.5)	10.75 (3.7)	10.58 (3.5)	10.70 (3.7)

*GUG* get up and go test

^a^Numeric rating scale (range 0–10)

^b^Seconds

**Table 4:** Statistical evaluation of the outcome measures by the comparisons

	Brace vs. no brace	*P* value	Tight brace vs. no brace	*P* value	Non-tight brace vs. no brace	*P* value	Non-tight vs. tight brace	*P* value
Outcomes, *B* (95% CI)^a^								
Pain, level walk	−0.60 (−0.97–0.23)	0.001*	−0.60 (−1.01–0.06)	0.028*	–0.60 (−1.01–0.07)	0.026*	−0.24 (−0.77 0.29)	0.37
Pain, perturbed walk	−0.80 (−1.11–0.43)	<0.001**	−0.75 (−1.31–0.02)	0.012*	−0.86 (−1.36–0.36)	0.001*	−0.21 (−0.44 0.30)	0.41
10 m walk test	−0.23 (−0.31–0.13)	<0.001**	–0.22 (−0.33–0.10)	<0.001**	−0.23 (−0.31–0.14)	<0.001**	0.11 (0.01 0.20)	0.03*
GUG test	−0.23 (−0.38–0.07)	0.004*	−0.08 (−0.25 0.08)	0.335	−0.38 (−0.57–0.17)	<0.001**	0.12 (−0.03 0.27)	0.13
Outcomes, OR (95% CI)^b^								
Knee instability, level walk	0.41 (0.23 0.66)	0.002*	0.37 (0.19 0.68)	0.002*	0.46 (0.22 0.93)	0.018*	1.06 (0.65 1.73)	0.79
Knee instability, perturbed walk	0.36 (0.22 0.59)	<0.001**	0.34 (0.18 0.62)	0.001*	0.39 (0.22 0.67)	0.001*	1.15 (0.66 2.0)	0.59
Knee confidence, level walk	0.45 (0.31 0.65)	<0.001**	0.48 (0.31 0.74)	0.001*	0.42 (0.27 0.65)	<0.001**	1.07 (0.75 1.51)	0.69
Knee confidence, perturbed walk	0.56 (0.38 0.82)	<0.001**	0.62 (0.35 0.77)	0.004*	0.63 (0.40 0.96)	0.033*	0.97 (0.60 1.56)	0.91

^a^Beta (B) coefficients (95% CI): negative value indicates reduced mean value of the outcome measure when wearing a brace

^b^Odds ratios (OR) (95% CI): value <1 indicates reduced chance of self-reported knee instability and lack of confidence in the knees when wearing a brace

*Significant at a *P* value <0.05; **significant at a *P* value <0.001

Whether the brace was non-tight or tight, the effects were similar (Table 4, columns 3 and 4). There was no difference in effects between a non-tight and tight brace, except for the 10-m walk test. Wearing a tight brace significantly reduced the time to complete the 10-m walk test in comparison with wearing a non-tight brace (*B* − 0.11, *P* = 0.03) (Table 4, column 5).

To determine whether the beneficial effects were in most participants or predominantly due to large effects in a few, the effect of wearing a soft brace on the outcome measures has been calculated per individual and presented on the histograms. The distribution of the individual effects per outcomes was fairly normal indicating that the overall beneficial effects are not attributable to large effects in a few participants [33].

## Discussion

In this study, we found that a soft brace reduced activity limitations and pain, and improved self-reported knee stability and knee confidence in patients with knee OA. We did not find significant differences between a non-tight and a tight brace, with the exception of performing the 10-m walk test. The results support the use of a soft brace as an efficacious treatment option in the immediate management of patients with knee OA.

This is the first high-quality study reporting on the immediate effect of wearing a soft brace, on pain, activity limitations, and self-reported knee instability, and knee confidence in patients with knee OA. The results of the recent systematic review on soft bracing in knee OA [6] showed that the quality of previous studies [2, 3, 15] ranged from low [3, 15] to fair [2] according to the Downs and Black scale [34]. If our study is scored according to the same criteria [34], the study is scored as a good-quality study (i.e., study quality percentage 68%). Compared to previous studies, our study received a higher score in the Downs and Black scale in statistical analysis, representativeness of the study population, and reporting on the facilities where patients were assessed.

The size of the effect on pain in our study is comparable to those observed in previous studies [2, 3, 15]. Bryk et al. [2] observed a 0.6 mm reduction in the visual analog scale (VAS) for pain during the Stair Climb Power Test (SCPT), while Schween et al. [3] and Hassan et al. [15] reported a similar decrease in the VAS for pain during level walking. We observed a 0.6-point (95% CI −0.97–0.23) and 0.8-point (95% CI −1.11–0.43) decrease in pain on the NRS for level and perturbed walking, respectively. This effect is close to the 1-point threshold for the minimal clinically important difference for NRS pain [35].

To our knowledge, only Bryk et al. [2] used performance-based physical tests to evaluate the immediate effect of a soft brace on activity limitations in patients with knee OA. Our study confirms their findings [2]. We found that wearing a soft brace significantly reduced time to complete both the 10-m walk and GUG tests by 0.23 s (95% CI −0.31–0.13 and −0.38–0.07, respectively). In the absence of information on the minimal clinically important change in these measures, the clinical relevance of these effects cannot be evaluated. Furthermore, these effects should be viewed in the light of this being a laboratory-based study and the short duration of brace use. Further research on generalization to functioning in daily life and the clinical relevance of these effects in daily life is indicated.

Previously, only one study had observed that a soft brace improved self-reported knee instability [3], while the effect on knee confidence had not been studied before. The present study confirms that a soft brace improves self-reported knee stability, and is the first study to show that a soft brace improved confidence in the knees, in patients with knee OA. These results are noteworthy, given that self-reported knee instability has been reported in the majority (>60%) of patients with knee OA [9, 14], and that it may affect their quality of life [9, 25]. Knee confidence may influence decisions about participation in physical activity. Lack of confidence may force patients with knee OA to alter their daily activities, to avoid knee buckling and pain [36]. A soft brace may therefore indirectly influence daily activities via the improvement of knee instability and knee confidence.

This is the first study using external perturbations on the treadmill to evaluate the effect of a soft brace on pain, and on self-reported knee instability and knee confidence. External perturbations provide a more stimulating and challenging environment for knee stability [37], and simulate situations in which patients might experience increased knee instability and lack of confidence. Knee OA could affect the ability of the neuromuscular system to execute appropriate commands in response to external challenges, resulting in joint instability [37]. It is therefore important to show that, even when participants were subjected to external perturbations, the effect of wearing a soft brace on pain and self-reported knee instability and knee confidence continued.

The second aim of this study was to assess the difference in effect between a non-tight and a tight brace. We hypothesized that a non-tight brace would elicit stronger effects than a tight brace. This hypothesis was based on a study reporting that a non-tight brace reduced pain and improved postural sway more than a tight brace [15]. Hassan et al. [15] speculated that a non-tight brace provided more recurrent stimuli, by allowing movement between the brace and the skin, and thus elicited continuous response from cutaneous receptors. By contrast, a tight brace might provide constant pressure, to which skin mechanoreceptors adapt. Our hypothesis was not confirmed. Differences in the type of the brace may be an explanation. Hassan et al. [15] used a tubigrip elastic bandage, whereas we used a soft brace specifically designed for the knee joint. More importantly, we are unaware whether the level of pressure exerted on the skin by our soft brace was comparable to that used by Hassan et al. [15]. There is therefore a need to develop a reliable and valid measure of tightness.

Because a soft brace is an efficacious intervention, there is a need to understand the mechanisms underlying the effect. Soft braces might reduce self-reported knee instability by altering proprioceptive feedback [15] and enhancing muscle activity [38]. Mild compression provided by a soft brace [2, 39] might result in an improved sense of joint stability and greater confidence in the knee joint [10]. It has been suggested that soft braces reduce joint contact forces, due to less muscle co-contraction [3, 40]. Another mechanism could be an increase in skin temperature [39, 40]. Heat therapies are widely used in clinical practice to obtain analgesia, decrease muscle spasm, and encourage regional blood flow [41]. Thus, a soft brace, by providing warmth to the skin, may lead to less muscle contracture and therefore to reduced knee joint compressive forces. Finally, a placebo effect is a mechanism that should be considered [4, 42]. To better understand how soft braces work in knee OA, studies exploring the potential mechanisms underlying the observed effects are warranted.

Some limitations should be acknowledged. First, it was not possible to blind participants to the brace; however, it should be noted that blinding to the intervention in this type of studies is generally not possible. Second, our study provided information on effect in the laboratory. Thus, we do not know whether the observed effects can be generalized to the daily life of patients with knee OA. In light of limited evidence in this area, this should be a priority for future research in order to determine whether wearing a soft brace has long-term effects in daily life in patients with knee OA. Finally, assessment of the outcomes was performed first without a brace and then with a brace. This order may have led to bias because of possible learning effects.

## Conclusions

In conclusion, the results of this study indicate that a soft brace is an efficacious intervention, targeting pain, activity limitations, and self-reported knee instability and knee confidence in the immediate-term in patients with knee OA. Although the results can potentially impact clinical practice, we acknowledge that studies on the generalization to functioning in daily life are required to support a soft brace as an established treatment option.

## Abbreviations

ACR: American College of Rheumatology
AMS-OA: Amsterdam Osteoarthritis cohort
BMI: Body mass index
CI: Confidence interval
GEE: Generalized estimating equations analysis
GUG: Get up and go test
ICC: Intraclass correlation coefficient
K&L: Kellgren & Lawrence
KL: Kellgren–Lawrence score
KOOS: Knee Osteoarthritis Outcome Score
NRS: Numeric rating scale
OA: Osteoarthritis
PFOA: Patello-femoral joint osteoarthritis
ROA: Radiographic knee osteoarthritis
SCI: Self-reported knee confidence
SCPT: Stair Climb Power Test
SD: Standard deviation
SKI: Self-reported knee instability
VAS: Visual analog scale
WOMAC: Western Ontario and McMaster Universities Osteoarthritis Index
